# Temperature dependent persistent luminescence: Evaluating the optimum working temperature

**DOI:** 10.1038/s41598-019-46889-z

**Published:** 2019-07-19

**Authors:** Jiaren Du, Olivier Q. De Clercq, Dirk Poelman

**Affiliations:** 1 LumiLab, Department of Solid State Sciences, Ghent University, Krijgslaan 281-S1, B-9000 Ghent, Belgium; 2 Center for Nano- and Biophotonics (NB-Photonics), Ghent University, B-9000 Ghent, Belgium

**Keywords:** Inorganic LEDs, Nanoparticles

## Abstract

Development of persistent luminescent materials has drawn continuous attention in recent years in view of their potential applications in the fields of security night-vision signage, *in vivo* bio-imaging and optical data storage. Currently, the normative evaluation of a new persistent luminescent material is focused on the light emission spectrum, the afterglow decay curve and the total duration time of the persistent luminescence. In this paper, we investigate the temperature dependent persistent luminescence in some well-known persistent phosphors and relate this to their thermoluminescence properties. The concept of the optimum working temperature is proposed as a new means for evaluating persistent phosphors. It is shown that there is a clear relation between the efficient temperature range of the afterglow output and the thermoluminescence glow curve. The experimental work is supported by simulations of thermoluminescence and afterglow characteristics. The concept of the optimum working temperature for persistent phosphors can be used as an evaluative criterion for applications in various working environments.

## Introduction

Persistent luminescent materials or long persistent phosphors (LPPs) are a group of luminescent materials possessing extraordinary energy storage ability and long-lasting emission after stopping the excitation. Development of persistent luminescent materials has drawn continuous attention because of their potential applications in the fields of emergency lighting signs, dials and security displays, night-vision signage, *in vivo* bio-imaging, dosimetry and optical data storage^1–3^. As one of the commercial LPPs for emergency lighting, the SrAl_2_O_4_:Eu^2+^, Dy^3+^ phosphor can give bright emission for more than 16 h (threshold value of 0.32 mcd/m^2^) at room temperature (RT) after removal of the excitation source^4,5^. Significant achievements have been made in the visible region and some representative LPPs emitting primary colors are SrAl_2_O_4_:Eu^2+^, Dy^3+^ with green emission^5,6^, CaAl_2_O_4_:Eu^2+^, Nd^3+^ with violet emission^7,8^, Sr_2_MgSi_2_O_7_:Eu^2+^, Dy^3+^ with blue emission^9^ and both CaS:Eu^2+^, Dy^3+^  ^10^ and Y_2_O_2_S:Eu^3+^, Mg^2+^, Ti^4+^ with red emission^11^. Near-infrared (NIR) persistent luminescent nano-particles are of particular interest for *in vivo* imaging, due to their merits of autofluorescence-free detection and high sign-to-noise ratio^3^. As a result, much effort has been spent to develop LPPs from the NIR emitting family. Up to now, there are growing numbers of persistent phosphors for NIR emission, with the representative ones including Zn_3_Ga_2_Ge_2_O_10_:Cr^3+^ ^2^, LiGa_5_O_8_:Cr^3+^ ^12–14^, La_3_Ga_5_GeO_14_:Cr^3+^ ^15,16^, LaAlO_3_:Mn^4+^ ^17,18^. The experimental material research has allowed the improvement of the LPPs in order to obtain a sufficiently strong and long lasting luminescence.

Persistent phosphors consist of an inorganic host matrix and activating dopant ions. The activator or dopant inside the host provides luminescence upon excitation and the emission color can be tuned as a function of the dopants and the host material^19,20^. The afterglow duration time can range from minutes to several hours after excitation mostly by ultraviolet or blue light, and is typically governed by co-dopants, intrinsic defects, or dopant-related defects^21,22^. These trapping defects can store charge carriers that are released from the excited state of the activator and gradually deliver these charge carriers over minutes to days, depending on the nature of the defects and the ambient temperature. In general, both emitters and traps play an important role in the persistent luminescence. Basic mechanisms for persistent luminescence have been suggested. However, the exact trapping and detrapping mechanism of the persistent luminescence is still under discussion^4,19,20,22^.

Currently, the normative evaluation of a new persistent luminescent material is focused on the emission spectra, the afterglow decay curves and the duration time of persistent luminescence. The spectral distribution during the afterglow process is an important evaluation criterion for a particular application and it determines the practical application of the LPPs. For example, the phosphors with persistent NIR emission after stopping excitation are very promising for advanced *in vivo* imaging, while green emission, close to the maximum eye sensitivity, is ideally suited for emergency lighting^23,24^. The afterglow decay curve is commonly applied to evaluate and compare LPPs. Useful information of traps can be obtained from the shape of the afterglow profile and it has been suggested that absolute intensity units should be used for specifying the afterglow instead of arbitrary units, in order to be able to make comparison between measurements and samples. For instance, units cd/m^2^ can be used for visible emission, whereby 0.32 mcd/m^2^ is often used as threshold for defining the afterglow time. For IR emission, this should be replaced by the equivalent radiometric unit mW/m^2^/sr. Hereby, 10^−3^ mW/m^2^/sr is roughly equivalent to 0.32 mcd/m^2^ ^25^. The duration time of persistent luminescence is a straightforward and standardized parameter to evaluate the new LPPs. It needs to be mentioned that all these standardized parameters are typically measured at room temperature, while the afterglow performance is temperature dependent and there could be a different optimum working temperature for a certain phosphor. As far as we know, a systematic exploration of the temperature dependence of persistent luminescence and its optimum working temperature has not been reported^26^. Insights into the relation between afterglow output and temperature yield very important information on the application potential of certain compounds and the way their performance can be optimized. In this paper, we propose the concept of the optimum working temperature as a new criterion for evaluating persistent phosphors.

Thermoluminescence (TL) measurements are effective to investigate the trapping behavior in persistent phosphors. Information on the amount of charge carriers and insights into the trap depth distribution inside these persistent phosphors can be obtained from TL glow curves^27^. In this work, TL glow curves are investigated for the prediction of the efficient afterglow output. The temperature dependent persistent luminescence is studied in some well-known persistent phosphors with thermoluminescence analysis. The persistent phosphors are Sr_2_MgSi_2_O_7_:Eu^2+^, Dy^3+^ (SMSO), SrAl_2_O_4_:Eu^2+^, Dy^3+^ (SAO), Sr_4_Al_14_O_25_:Eu^2+^, Dy^3+^ (SAO25), CaAl_2_O_4_:Eu^2+^, Nd^3+^ (CAO), Y_2_O_2_S:Eu^3+^, Mg^2+^, Ti^4+^ (YOS), CaS:Eu^2+^, Dy^3+^ (CaS) and LaAlO_3_:Mn^4+^, Na^+^ (LAO) phosphors. We show that there is a clear relation between the optimum afterglow temperature range and its corresponding thermoluminescence glow curve in commonly used persistent phosphors. A strategy for evaluation of the optimum working temperature for persistent phosphors is thus proposed through the TL measurements. The experiments are supported by simulations of thermoluminescence and afterglow characteristics. The experimental TL data can be described and afterglow properties at various temperatures can be predicted using the well-known Randall-Wilkins first-order expression of a single glow peak model. Realizing the optimum working temperature of persistent phosphors, appropriate candidates can be chosen for a certain temperature environment. The approach used in this paper is easily transferable to other new persistent phosphors for better practical applications.

## Results

**Figure 1:**
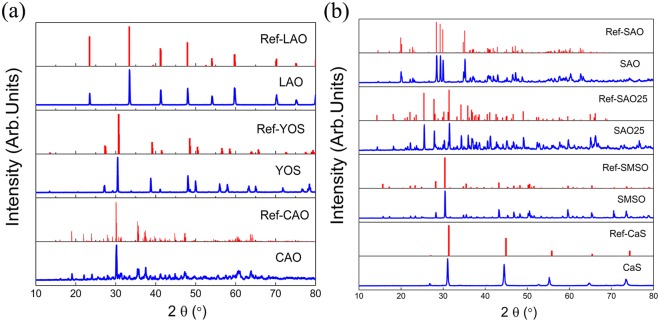
Measured XRD patterns (blue curves) of the phosphors investigated and standard XRD patterns (red curves) of the host crystal structures.

The measured XRD patterns and their corresponding reference patterns of LAO, YOS, CAO, SAO, SAO25, SMSO and CaS phosphors are shown in Fig. 1. All XRD patterns are well indexed to the standard data and the substitution of the doping atoms in the crystal lattice does not have a detectable influence on the XRD patterns. The measured XRD patterns match the structures of the corresponding host lattices (CAO-ICSD No.000260 ^28^, YOS-ICSD No.067503 ^29^, SMSO-ICSD No.031308 ^30^, SAO-ICSD No.026466 ^31^, SAO25-ICSD No.027744 ^32^, CaS-ICSD No.619527 ^33^ and LAO-ICSD No.153821 ^34^), without traces of other crystalline phases.

**Figure 2:**
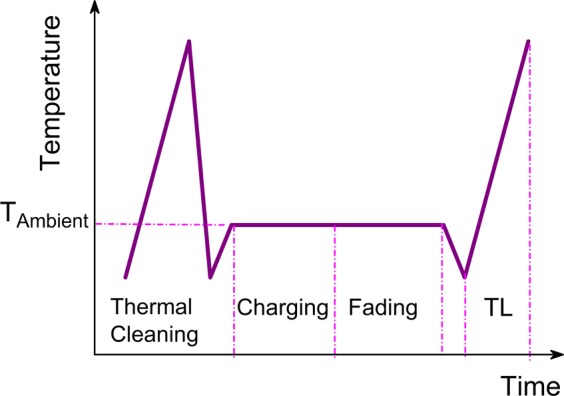
Schematic view of the TL experimental procedure to investigate the temperature dependence of the persistent luminescence.

**Figure 3:**
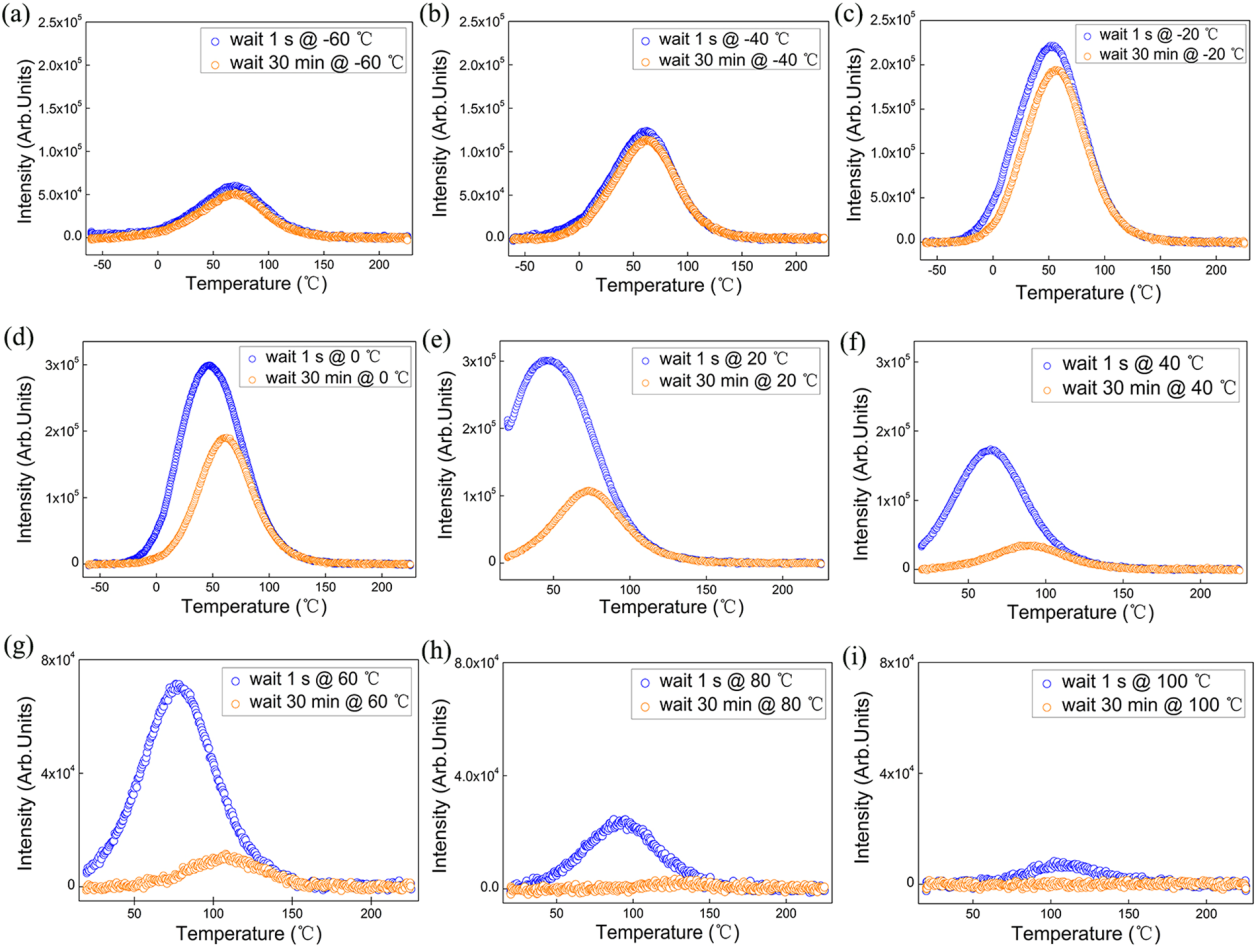
(**a**–**i**) TL glow curves of Sr_2_MgSi_2_O_7_:Eu^2+^, Dy^3+^ (SMSO) at various charging temperatures for different fading times (after irradiation for 10 min at charging temperature from −60 °C to 100 °C, fading time was chosen as 1 s or 30 min, and TL curves were collected with a constant heating rate of 30 °C min^−1^).

In order to investigate the temperature dependence of the persistent luminescence of these phosphors, a dedicated TL experiment was conducted as shown in Fig. 2 (details in Materials and methods). A series of these TL experiments with different fading times and temperatures were performed. Following the experimental procedure, the TL results of SMSO for various charging temperatures and different fading times are illustrated in Fig. 3(a–i). SMSO is taken as an example and measurements on other phosphors mentioned are also performed in this work (see Figs S1–S6). The color codes in all these individual figures are the same: The blue TL glow curves represent the total light emission from the SMSO phosphor after 1 second fading time and the orange TL glow curves show the total light emission during the TL experiment after 30 minutes fading time. The charging or fading temperature is in the range from −60 °C to 100 °C as shown in Fig. 3(a–i). As the temperature rises, detrapping of the captured charge carriers and recombination take place, thus the intensity of TL glow curve initially increases. With further increasing the temperature, the probability of escape becomes much higher and the trapped charge carriers from trapping levels are released. Thus, the TL intensity reaches a maximum (the occurrence of the peak maximum). As the amount of charge carriers trapped inside the phosphor becomes depleted, the TL intensity finally decreases and no luminescence can be detected anymore. The total light emission of the TL glow profile after 1 second fading can be regarded as proportional to the total amount of trapped charge in the LPPs. The difference between the total light output during the TL measurements after a 1 second and 30 minutes fading time is approximately equivalent to the afterglow output of the persistent phosphor at each temperature, in a time frame of 30 minutes. Thus, the influence of temperature on the afterglow in a time frame of 30 minutes can be evaluated, using the above-mentioned TL procedure. Of course, longer fading times could be used, if the total afterglow during much longer times would be relevant. From Fig. 3(a), there is almost no difference between the two TL glow curves with different fading times because there is no efficient detrapping after charging at −60 °C. The initial intensity of the TL glow peak is relatively low and excitation at lower temperature does not lead to efficient trap filling. At this temperature range, the afterglow output from the phosphor is constrained by the thermal barrier and the limited efficiency of the trap filling. As the temperature increases (from −40 °C to 20 °C), more thermal energy is readily available and excitation is expected to fill traps more efficiently^6^. Therefore, the intensity of the TL glow peak increases gradually. Charge carriers trapped inside the phosphor release more energy with a longer fading time. In the temperature range from −40 °C to 20 °C, the afterglow output keeps increasing monotonically as shown in Fig. 3(b–e). More charges are trapped and released in the material, which plays a predominate role in the afterglow performance. Nevertheless, the position of the glow peaks is very similar, suggesting the same trapping center is filled in the SMSO phosphor, irrespective of charging temperature. The afterglow output reaches a maximum and the optimum working temperature of the SMSO phosphor is found around 20 °C. With a further increase of charging temperature (in Fig. 3(e–i)), the intensity of the TL glow peak drops because less traps are filled at elevated temperature. The charge carriers are also releasing their energy during the charging period and only the deeper traps remain filled at elevated temperature. As a result, in Fig. 3(e–i), a shift of the TL glow peak towards higher temperatures appears and the afterglow output decreases with further increasing temperature. After excitation at 100 °C (shown in Fig. 3(i)), we can only see the weak TL glow peak located around 100 °C, which indicates most trapped charge carriers are depleted and a very limited trap distribution remains in deeper traps. There is a large difference of the afterglow output in the same SMSO phosphor at different temperatures in Fig. 3, which indicates the persistent luminescence is a thermally activated process^6^, largely influenced by its working temperature.

**Figure 4:**
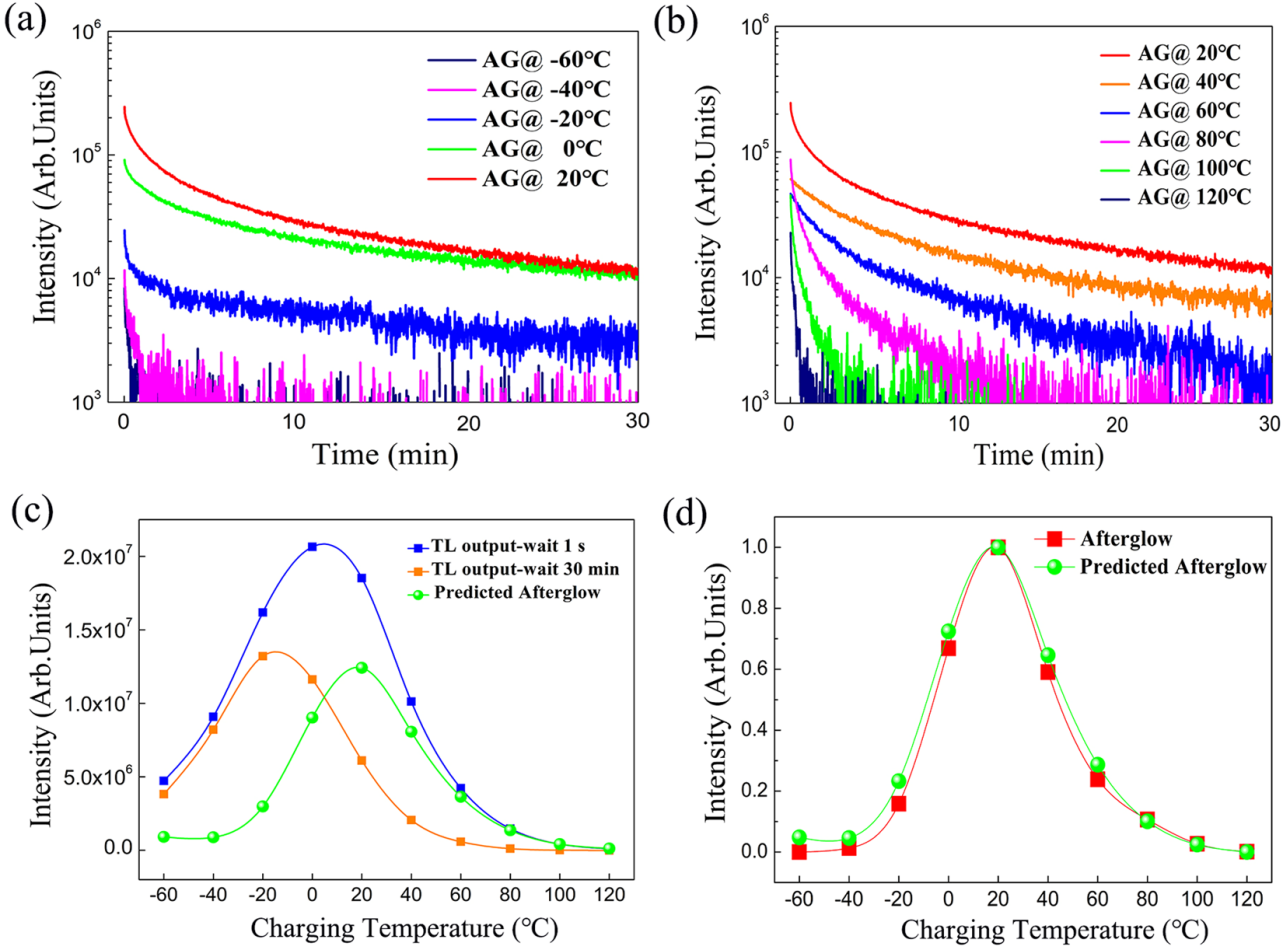
(**a**) Afterglow curves of SMSO phosphor measured for 30 min after 10 min excitation at each temperature from −60 °C to 20 °C. (**b**) Afterglow curves of SMSO phosphor measured for 30 min after 10 min excitation at each temperature from 20 °C to 120 °C. (**c**) Integrated light emission output during the TL measurement at various charging temperatures for 1 sec fading (blue curve) and 30 min fading time (orange curve). The predicted afterglow output of the SMSO phosphor (green curve) is extracted from blue and orange curves. (**d**) Comparison of the experimental afterglow profiles and the predicted afterglow output at various temperatures. The experimental afterglow data at various temperatures is integrated over 30 minutes and plotted as a function of temperature (the red curve).

Afterglow measurements at various temperatures were performed with the aim to compare with TL experiments. The afterglow intensity of SMSO phosphor as a function of time, measured at lower temperatures, from −60 °C to 20 °C, and at elevated higher temperatures, from 20 °C to 120 °C, are illustrated in Fig. 4(a),(b). The afterglow profiles are plotted in a semilogarithmic graph. The intensity of the afterglow curves is temperature dependent and reaches its ideal performance while the temperature is around 20 °C. To predict and evaluate the ideal temperature of the afterglow performance, the above-mentioned TL procedure (see Fig. 2) was also utilized. Figure 4(c) presents the integrated light emission output during the TL measurement (blue curves with 1 sec fading and orange curves with 30 min fading) after charging 10 min at temperatures from −60 °C to 120 °C. The predicted afterglow output of the SMSO phosphor (green curves extracted from blue and orange curves) was estimated from the thermoluminescence experiments, by subtracting the total TL output after 30 min fading from the integrated TL intensity after 1 sec fading. In addition, we also integrated the experimental afterglow data at various temperatures (from Fig. 4(a,b)) over 30 minutes and plotted it as a function of temperature (red curves as seen in Fig. 4 (d)). There is a good agreement between the experimental afterglow measurements and the predicted afterglow output extracted from a series of the TL experiments, the optimum working temperature of SMSO phosphor is evaluated to be around 20 °C. This result of multiple experiments confirms that 20 °C is the ideal working temperature of SMSO phosphors for practical application.

**Figure 5:**
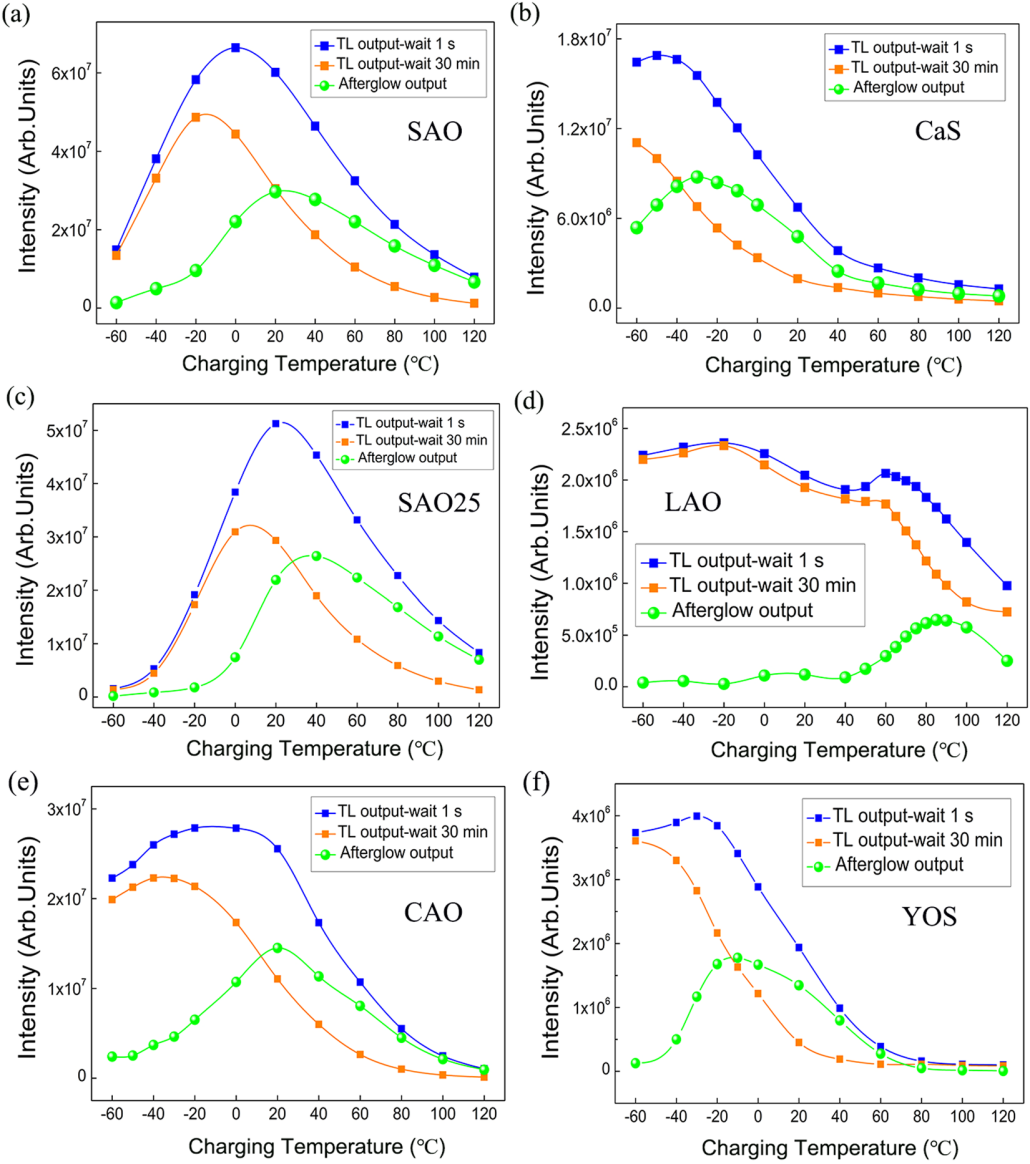
Blue curves: Integrated light emission during the TL measurement (with 1 sec fading) after charging 10 min at different temperatures, i.e., a measure for the total accumulated light emission from the phosphor or the total amount of filled traps after excitation. Orange curves: Integrated light emission during the TL measurement (with 30 min fading) after charging 10 min at different temperatures. Green curves: the extracted afterglow output from blue and orange curves at different temperatures. (**a**) SAO phosphor (**b**) CaS phosphor (**c**) SAO25 phosphor (**d**) LAO phosphor (**e**) CAO phosphor (**f**) YOS phosphor.

To evaluate the optimum working temperature for other compounds, this TL experimental procedure as described above was also applied to other persistent materials (SAO, SAO25, CAO, YOS, CaS and LAO). The same color codes were used in Fig. 5: Blue curves give the integrated light emission during the TL measurement (with 1 sec fading) after charging 10 min at different temperatures, which is regarded as a measure for the total accumulated light emission from the phosphor or the total amount of filled traps after excitation. Orange curves represent the integrated light emission during the TL measurement (with 30 min fading) after charging 10 min at different temperatures. Green curves are the extracted afterglow output from blue and orange curves at various temperatures, integrated over 30 minutes. It is clear that the predicted afterglow output is temperature dependent and the temperature dependent persistent luminescence relies on the nature of the phosphors. Different persistent phosphors show different optimum working temperature ranges: SAO and SAO25 phosphors have a good persistent luminescence performance around room temperature. It is worth mentioning that the red-emitting commercial CaS persistent phosphor exhibits an optimum working temperature around −30 °C. It indicates CaS could find its applications in a lower temperature environment and performs better there, than at elevated temperatures. On the other hand, the LAO phosphor gives its best NIR persistent luminescence at higher temperature side (around 90 °C), due to the higher trap depths in this material^35^.

**Figure 6:**
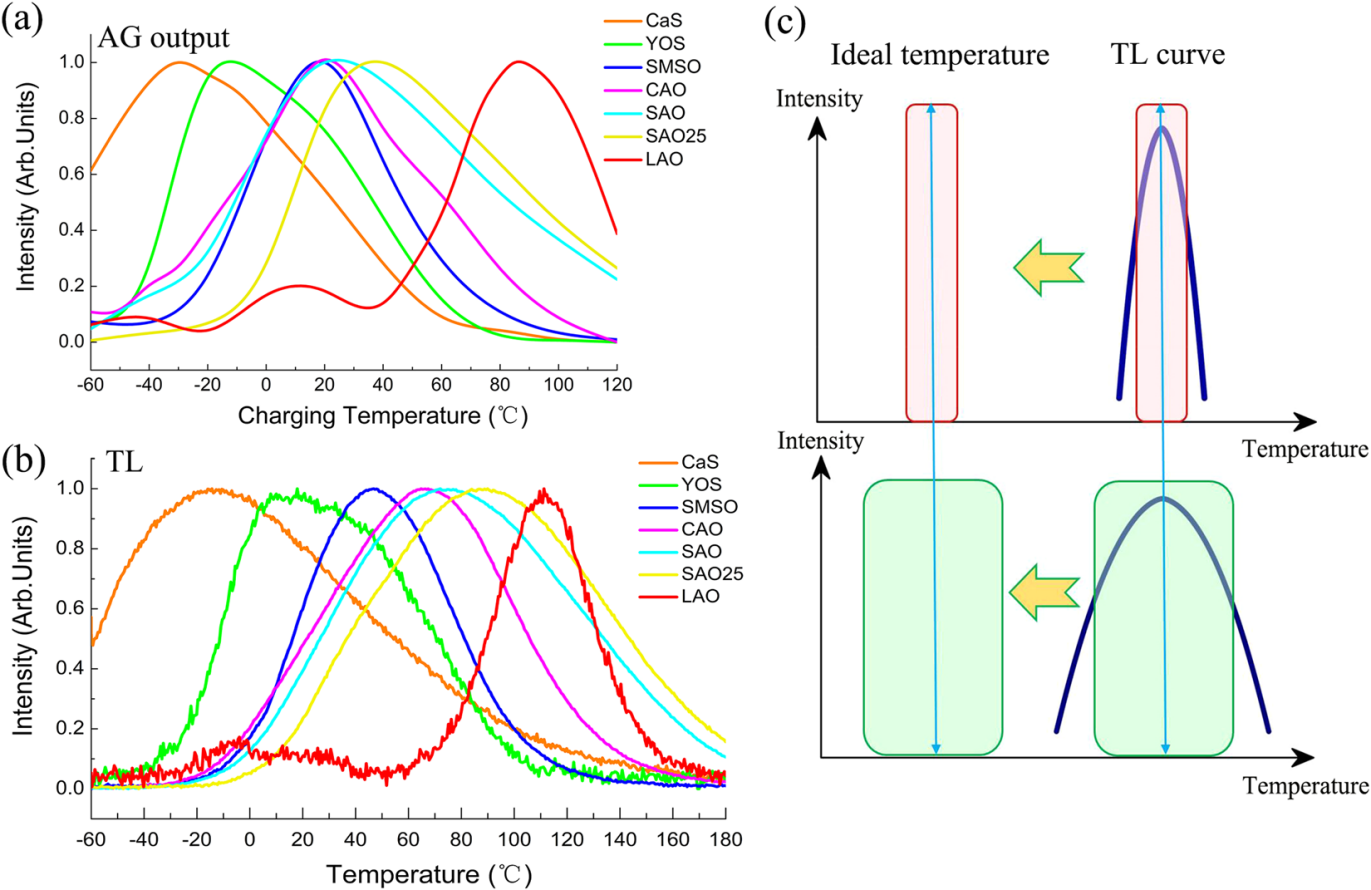
(**a**) Comparison of the normalized afterglow output of the different LPPs. (**b**) Comparison of the normalized TL glow curves of the different LPPs. (**c**) Schematic view of the relation of the optimum temperature range and TL glow curve.

The predicted afterglow output of the different LPPs is given as a function of temperature and their corresponding normalized TL glow curves are shown in Fig. 6 (a,b). A detailed comparison of the afterglow output and its corresponding TL glow curve of each LPP are shown in Fig. S7. There is a clear relation between the efficient temperature range of the predicted afterglow output and the thermoluminescence glow curve in these commonly used persistent phosphors. The shape of the afterglow output as a function of temperature and the relative position of the peaks behave in accordance with their corresponding TL glow curves, which is attributed to the nature of the traps inside the phosphor. The temperature dependent persistent luminescence of a phosphor is largely determined by its corresponding trap distribution and trap depths, which can be studied from the thermoluminescence curve as seen in Fig. 6(c). Compared with the normalized afterglow output of different persistent phosphors in Fig. S7, all the TL glow peaks are located at the right side (higher temperature side). In other words, the afterglow output or the best/ideal working temperature is located at the left side (lower temperature side) of its corresponding TL glow peak. In general, the temperature of the main TL peak relates to the temperature of the strongest intensity of the persistent luminescence and the optimum working temperature is located at the lower temperature side of the main TL peak. The trap distribution gives the range or the width of the optimum working temperature. A wide range of optimum working temperatures originates from a broad and continuous trap distribution of a phosphor. On the other hand, phosphors with a very narrow trap depth distribution show a smaller width of the TL glow peak, resulting in a narrower range of the ideal working temperature. Therefore, the optimum working temperature range can be regarded as a reflection of the TL glow curve to lower temperature side. The central position of the TL glow curve is shifting to lower temperature side to form the ideal working temperature range and the width of this temperature range is dependent on the shape of its corresponding TL glow curve.

## Discussion

**Figure 7:**
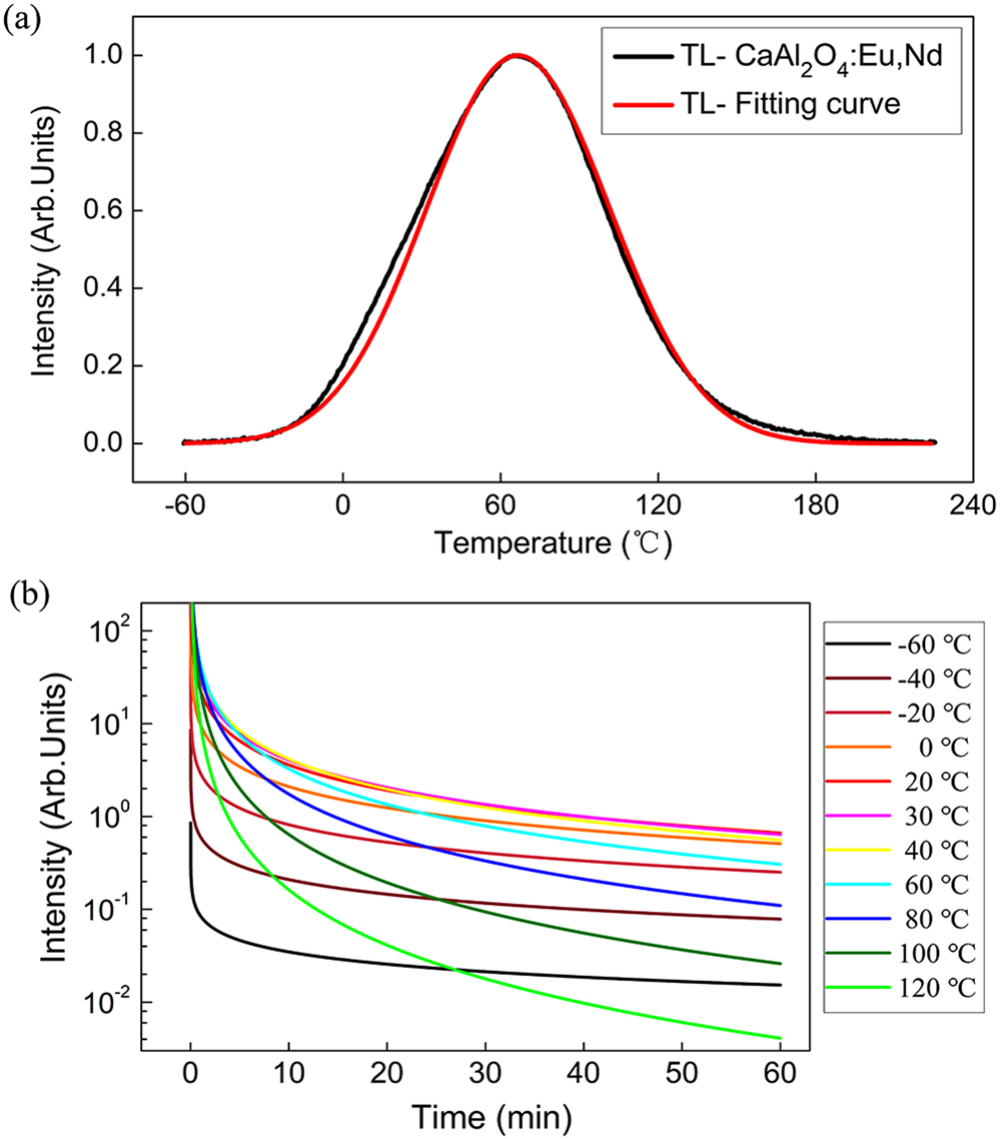
(**a**) TL glow curve of CAO phosphor and its corresponding fitting curve. (**b**) Prediction of afterglow profile of CAO phosphor at different temperatures.

To further verify the ideal temperature range of the persistent luminescence proposed by the experimental procedures, a simulation of the TL glow curve and the afterglow profile at various temperatures was made. The detrapping behavior was assumed to follow the first-order kinetics, thus negligible retrapping during the heating stage was assumed^27,36^. The Randall-Wilkins first-order expression of a single glow peak was applied as described in the methods section^37,38^. Computerized TL glow curve fitting was used to extract the relevant trapping parameters from the experimental TL measurements. Extracted fit parameters of these phosphors are given in Table 1. The value for the frequency factor *s* and linear heating rate were kept constant at 10^12^ s^−1^ and 0.5 K s^−1^ respectively. The TL glow curve of the CAO phosphor and its corresponding fitting curve are illustrated as an example in Fig. 7(a). By fitting the experimentally recorded data, afterglow properties at various temperatures can be predicted as shown in Fig. 7(b). In this work, the afterglow simulation at various temperatures was applied to all these phosphors as shown in Fig. S8. In accordance with the TL procedure, the calculated afterglow profiles were integrated over the first half hour time and the data were plotted as a function of temperature to obtain the ideal afterglow temperature range.

**Table 1:** Extracted fit parameters to describe the TL data.

*Phosphor*	*E*_*G*_ (*eV*)	*σ*_*G*_ (*eV*)
SMSO	0.865	0.075
CAO	0.915	0.091
YOS	0.795	0.090
SAO	0.943	0.123
SAO25	0.961	0.119
LAO	1.037	0.049
CaS	0.695	0.130

The value for the frequency factor s and linear heating rate were kept constant for all seven phosphors at 10^12^ s^−1^ and 0.5 K s^−1^.

**Figure 8:**
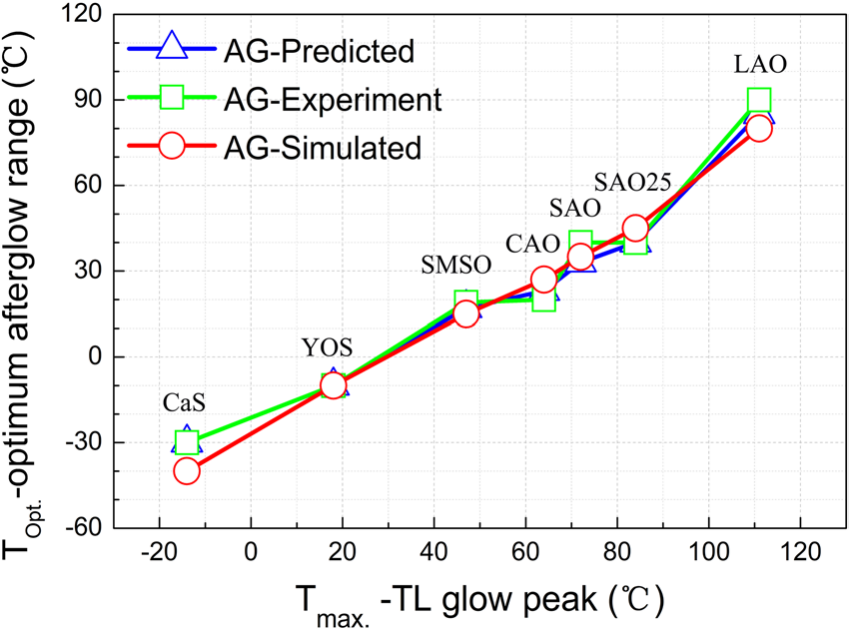
Comparison of the optimum afterglow temperature (T_opt._) and the temperature of the TL glow peak (T_max._) from the afterglow simulation (AG-Simulated), experimental afterglow measurements (AG-Experiment) and the predicted afterglow output from TL measurements at various temperatures (AG-Predicted). Each data point stands for a specific phosphor, namely, CaS, YOS, SMSO, CAO, SAO, SAO25, and LAO phosphor from left to right.

A comparison of the optimum afterglow temperature (T_opt._) and the temperature of the TL glow peak (T_max._) is presented for all the phosphors in Fig. 8. The afterglow simulation, the predicted afterglow output from experimental TL measurements and afterglow output from experimental afterglow measurements are illustrated and compared. There is a good agreement among the simulated, predicted and experimental afterglow profiles and the relation between the optimum afterglow temperature range and its corresponding TL glow peak is coherent from all these methods. The optimum working temperature is always located at the lower temperature side of its corresponding TL peak from both simulation and experimental methods. The good correspondence indicates the nature of trap states (trap depth and trap level distribution) inside the phosphor is responsible for the temperature dependent persistent luminescence. It should be noted that the same TL settings should be applied in order to obtain a reasonable and quantitatively comparable optimum working temperature, since the TL parameters (such as heating rate) have a big influence on the TL glow curve. For evaluating the ideal working temperature, the estimation from TL glow curve is more practical and timesaving than the trial-and-error method of afterglow measurement at various temperatures considering a single afterglow measurement requires dozens of hours to obtain a complete decay curve. Hence, the proposed concept of the optimum working temperature and the experimental procedures are reasonable, giving a new and concise means for evaluating persistent phosphors.

It is very interesting to shed light on the temperature dependent persistent luminescence of a new LPP. From an application point of view, a better grasp of the influence of the temperature is useful. For applications at room temperature, SAO, SAO25 and SMSO are very good candidates, which currently are extensively used. At relatively low temperature, these candidates are not favorable since their afterglow performance is worse at low temperatures. Obviously, to make best use of the CaS-based persistent phosphor, a lower temperature environment is ideal. Furthermore, in the case of the LAO phosphor, it only performs well at elevated temperature. Therefore, the new evaluative criterion - the optimum working temperature - is proposed to give a better description and evaluation of the persistent phosphors in practical applications. The proposed method describes the ideal temperature range for maximum emission in the first half hour and the method could easily be extended to much longer fading times. To avoid the time consuming trial-and-error of extensive afterglow tests, a measurement of a single TL glow curve of a persistent phosphor thus is a very fast and easy method to assess both the expected total afterglow output and the ideal working temperature range for any application. The concept of the optimum working temperature as a new evaluative criterion for persistent phosphors is applicable to further exploration and development of persistent phosphors.

Temperature dependent persistent luminescence was studied in a number of persistent phosphors with thermoluminescence analysis. We showed that there exists a clear relation between the efficient temperature range of the afterglow output and the thermoluminescence glow curve in persistent phosphors. The concept of the optimum working temperature is proposed as a new means for evaluating persistent phosphors. Also, the temperature dependent persistent luminescence of a phosphor is largely determined by its corresponding trap distribution and trap depth. The experimental work is supported by simulations of thermoluminescence and afterglow characteristics. It is expected the evaluative criterion of the optimum working temperature for persistent phosphors can be used as guidance for applications in various working temperature environments.

## Materials and Methods

Several widely studied and commercialized persistent luminescent compounds (Glo.Tech.Intl.), namely Sr_2_MgSi_2_O_7_:Eu^2+^, Dy^3+^ (SMSO), SrAl_2_O_4_:Eu^2+^, Dy^3+^ (SAO), Sr_4_Al_14_O_25_:Eu^2+^, Dy^3+^ (SAO25), CaAl_2_O_4_:Eu^2+^, Nd^3+^ (CAO), Y_2_O_2_S:Eu^3+^, Mg^2+^, Ti^4+^ (YOS), CaS:Eu^2+^, Dy^3+^ (CaS) were used as well as the LaAlO_3_:Mn^4+^, Na^+^ (LAO) phosphor synthesized in our lab^18,35^.

The crystal structures of the studied powders were verified using powder X-ray diffraction (XRD) measurements on a Siemens D5000 diffractometer (40 kV, 40 mA, Bruker) using Cu Kα1 radiation (λ = 0.154 nm). The XRD data were collected in the range 2θ from 10*◦* to 80*◦* at room temperature with a step time of 1.5 second and step size of 0.02*◦*. The measured XRD patterns were compared with the standard data from the structures of the corresponding host lattices.

A small lab-built vacuum chamber with a well-characterized cooling and heating stage was used for TL measurements^6^. Identical size thin pellet samples (thickness ca. 2.0 mm, diameter 5.0 mm) were used and these samples were in good thermal contact with the heat exchanger by using thermally conductive adhesive. The excitation source for the charging was a monochromated 300 W Xenon arc lamp (260 mm, f 3.9 monochromator). The fiber-coupled monochromator in combination with light source allows strong and direct monochromatic excitation light towards the sample. The light emitted from the samples was guided by an optical fiber and collected using a ProEM1600 EMCCD camera connected to a 300 mm monochromator (Princeton Instruments). At a certain ambient temperature *T*_*Ambient*_, the sample was charged by constant irradiation intensity with monochromatic light of an appropriate wavelength (see Table 2). The charging duration was 10 minutes in all cases, which is sufficient to obtain a complete trap filling and strong enough TL readout intensity. Subsequently, at the same temperature *T*_*Ambient*_, the sample was kept in the dark for a variable fading period. Then the sample was cooled and a TL measurement was performed up to the maximum temperature (225 °C) using a constant heating rate β (β = 30 °C min^−1^). This moderate heating rate was chosen and tested to improve the signal-to-noise ratio and prevent temperature differences between the heating plate and the phosphor studied, which is beneficial for the precision and repeatability of the measurements. A fixed cooling rate of 50 °C min^−1^ was employed for each TL cooling process. In addition, the two starting temperatures (below 0 °C: starting temperature of −60 °C; above 0 °C: starting temperature of 20 °C) for TL recording were used to limit the loss of energy from a long cooling process and improve the precision of the TL readout. The maximum 225 °C was fixed and no higher temperature was applied in order to restrain the thermal quenching (TQ) effects which may suppress the TL behavior at higher temperatures, but these trapped charges could still contribute to the afterglow which occurs at lower temperatures^39^. A series of these TL experiments were performed, each one having a different fading time (waiting 1 second or 30 minutes) and a different temperature *T*_*Ambient*_ in the range from −60 °C to 120 °C with an interval of 20 °C. Prior to each TL experiment, a thermal cleaning was conducted by heating up to 225 °C, to assure all relevant traps were empty at the start of experiment. A schematic view of the TL experimental procedure is shown in Fig. 2, including thermal cleaning, charging, fading and TL stages.

**Table 2:** Excitation wavelength and integrated emission ranges.

*Phosphor*	*λ*_*ex*_ (*nm*)	*λ*_*em*_ (*nm*)
SMSO	365	400–600
CAO	326	400–550
YOS	325	540–730
SAO	435	450–700
SAO25	370	400–650
LAO	335	650–800
CaS	470	500–750

Afterglow decay curves at each temperature were collected with a ProEM1600 EMCCD camera attached to an Acton SP2300 monochromator (Princeton Instruments). The excitation source was a monochromated 300 W Xenon arc lamp (Oriel Instruments, Stratford, CT, U.S.A.) at a certain wavelength for 10 min at each temperature. Different characteristic emission spectra were collected and integrated according to the corresponding emission wavelength ranges of the different phosphors. The excitation wavelengths and integrated emission ranges were applied as shown in Table 2. These excitation and emission ranges are in line with literature reports^6,10,18,40–43^.

In order to simulate the thermoluminescence glow curves, the first-order expression of a trap depth distribution from the Randall-Wilkins method^37,38^ was applied.

1 $$I(T)={\int }_{0}^{\infty }\,\frac{s}{\beta }N(E){f}_{0}(T)\exp \{-\frac{E}{kT}\}\times \exp \{-\frac{s}{\beta }{\int }_{{T}_{0}}^{{T}^{\text{'}}}\exp \{-\frac{E}{kT^{\prime} }\}dT^{\prime} \}dE$$

The term *s* is the frequency factor or attempt-to-escape factor, which was considered as a constant value independent of temperature. The value of *s* was kept constant at 10^12^ s^−1^ of the order of the lattice vibration frequency. *β* is the constant linear heating rate (K s^−1^), and 0.5 K s^−1^ (30 °C min^−1^) was employed for all the TL measurements. *N(E)* is a continuous distribution in trap depths and several distribution shapes can be considered. In this work, we chose a Gaussian shape where the mean energy and the width of the Gaussian function are dependent on the material studied. *f*_0_*(T)* is the filling factor of the distribution and saturated filling of traps was assumed with *f*_*0  *_ = 1. *E* is the trap depth or activation energy which is needed to release the charge carrier (an electron or a hole) from the trap into the conduction band or the valence band. *k* is the Boltzmann constant with a value of 8.6173303 × 10^−5^ eV K^−1^. *T* is the absolute temperature in Kelvin. The expression *(1)* gives the TL intensity as function of temperature and describes the TL data of a single glow peak with the choice of expression for the trap distribution. The relevant Gaussian function with mean energy E_G_ and width *σ*_G_ can be extracted by utilizing this expression. It is worth noting that some simplifying assumptions are made to develop the analytical equation for first-order kinetics, such as negligible retrapping during the heating stage (assumption made by Randall and Wilkins^37,38^) and the quasi equilibrium assumption by Chen and McKeever^44^. Gaussian distributions were chosen as a reasonable assumption for a random incorporation of trap states^39,45^. Some other possible trap depth distributions have been investigated such as exponential^46,47^, uniform^48,49^ or binomial^50^ trap depth distribution models in literature, but for all selected materials in this work, the approximation used was found to yield good results.

The obtained parameters for the trap distribution from the TL glow curves were utilized as input for the simulation of the afterglow profile. The expected afterglow profile follows the equation of the first-order kinetics in the presence of a trap distribution^48^.

2 $$I(t)={\int }_{0}^{\infty }\,sN(E){f}_{0}(T){e}^{-E/kT}\exp [-st\,{e}^{-E/kT}\,]dE$$

The simulation of the afterglow at different temperatures was made for all the investigated phosphors. The effective working temperature region from the simulation results was further compared with experimental data.

## Supplementary information

Supporting information
