# Supplementary material for: Temperature dependent persistent luminescence: Evaluating the optimum working temperature
Source: Sci Rep. 2019 Jul 19;9:10517. doi: 10.1038/s41598-019-46889-z (PMC6642144; doi:10.1038/s41598-019-46889-z)
Supplement: Supplementary file 1 — Supporting information [file 41598_2019_46889_MOESM1_ESM.pdf]

Supporting Information

Temperature dependent persistent luminescence: Evaluating the optimum working temperature

Jiaren Du, Olivier Q. De Clercq, Dirk Poelman*

LumiLab, Department of Solid State Sciences, Ghent University, Krijgslaan 281-S1, Ghent, Belgium;

*Correspondence: Dirk.Poelman@ugent.be;

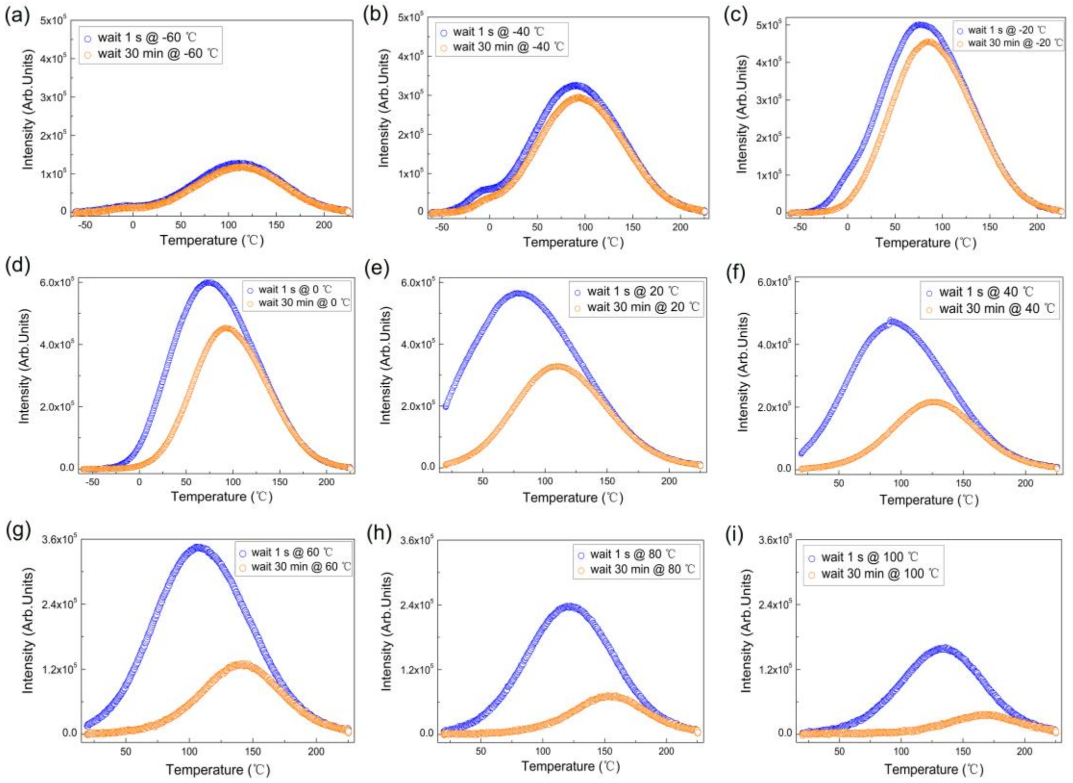

Figure S1. TL glow curves of $SrAl_2O_4:Eu^{2+}$, Dy^{3+} (SAO) at various charging temperatures for different fading times (after irradiation for 10 min at charging temperature from -60 °C to 100 °C, fading time was chosen as 1 s or 30 min, and TL curves were collected with a constant heating rate of 30 °C min^{-1}).

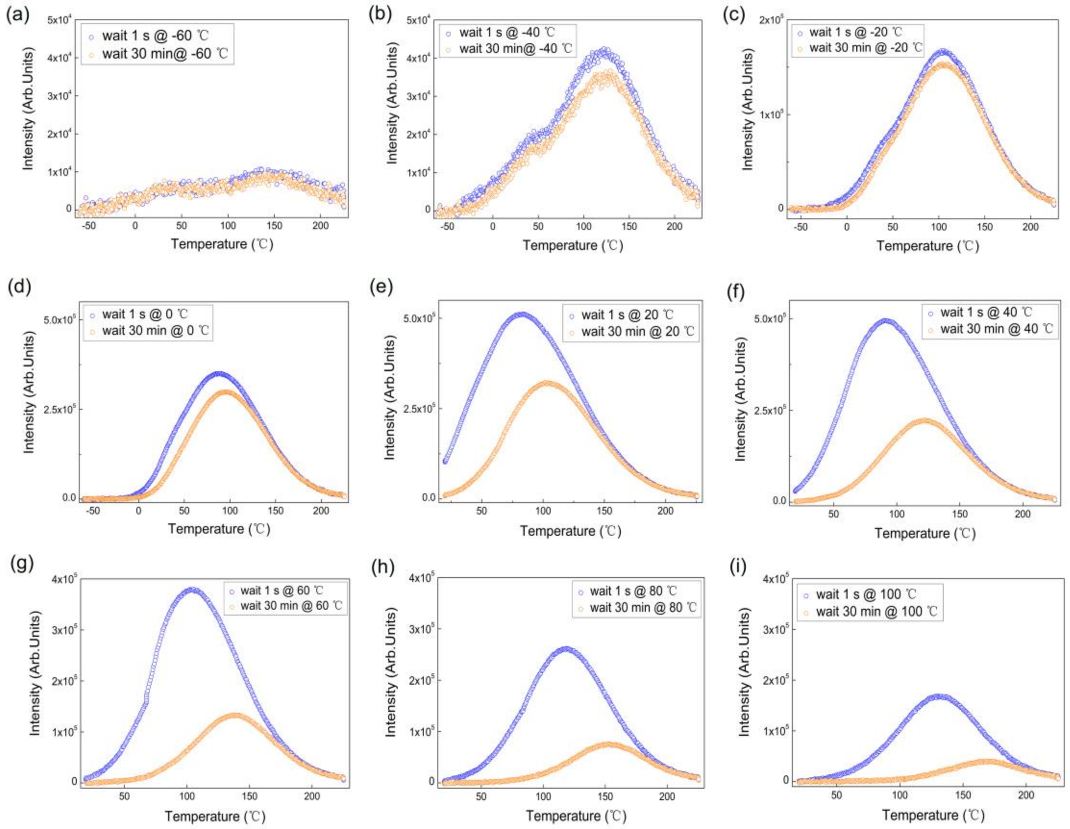

Figure S2. TL glow curves of $Sr_4Al_{14}O_{25}$:Eu^{2+}, Dy^{3+} (SAO25) at various charging temperatures for different fading times (after irradiation for 10 min at charging temperature from -60 °C to 100 °C, fading time was chosen as 1 s or 30 min, and TL curves were collected with a constant heating rate of 30 °C min^{-1}).

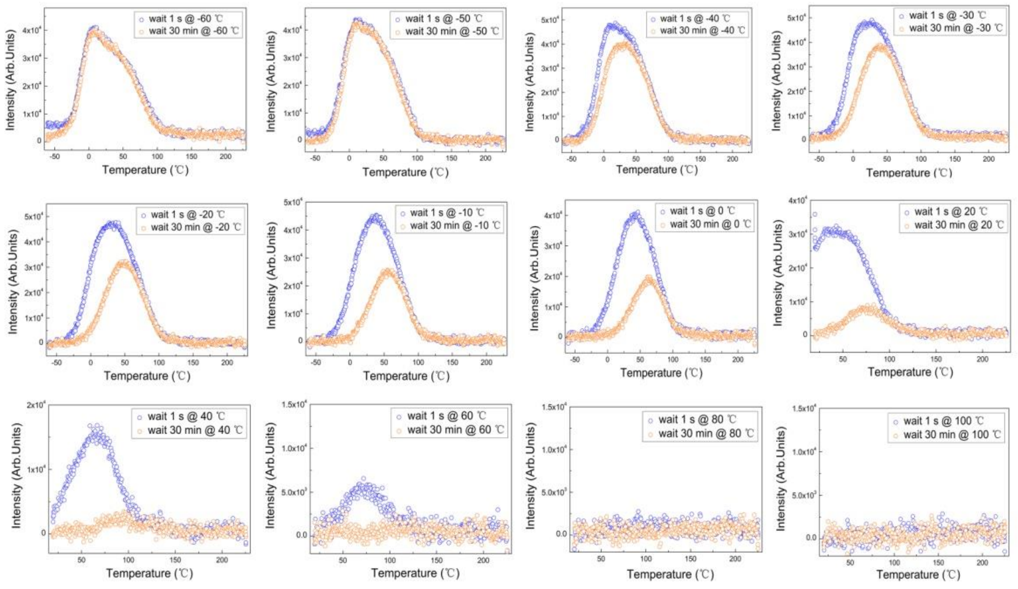

Figure S3. TL glow curves of $Y_2O_2S:Eu^{3+}$, Mg^{2+}, Ti^{4+} (YOS) at various charging temperatures for different fading times (after irradiation for 10 min at charging temperature from -60 °C to 100 °C, fading time was chosen as 1 s or 30 min, and TL curves were collected with a constant heating rate of 30 °C min^{-1}).

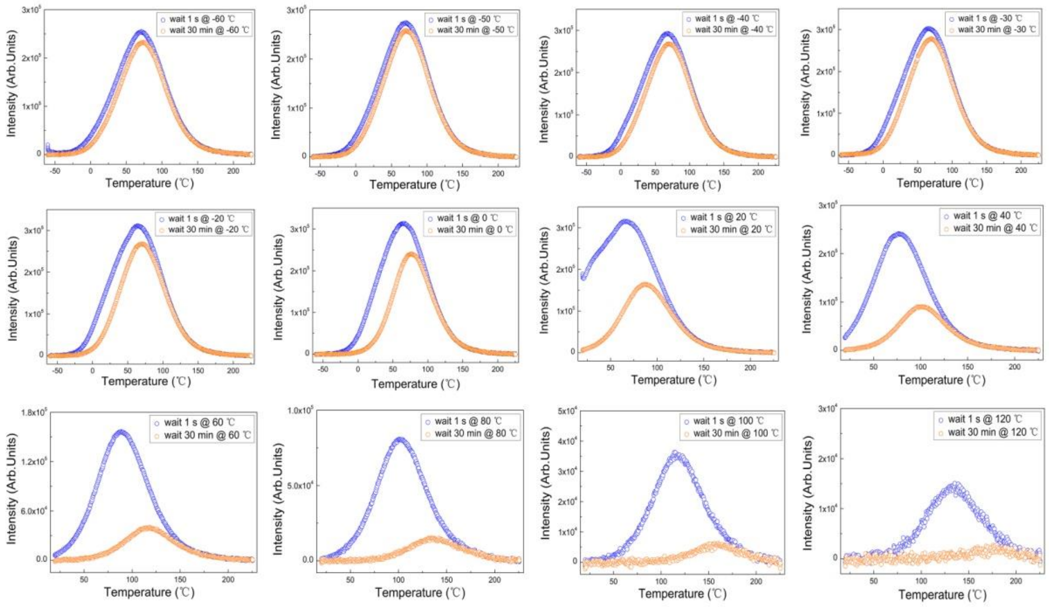

Figure S4. TL glow curves of $CaAl_2O_4:Eu^{2+}$, Nd^{3+} (CAO) at various charging temperatures for different fading times (after irradiation for 10 min at charging temperature from -60 °C to 120 °C, fading time was chosen as 1 s or 30 min, and TL curves were collected with a constant heating rate of 30 °C min^{-1}).

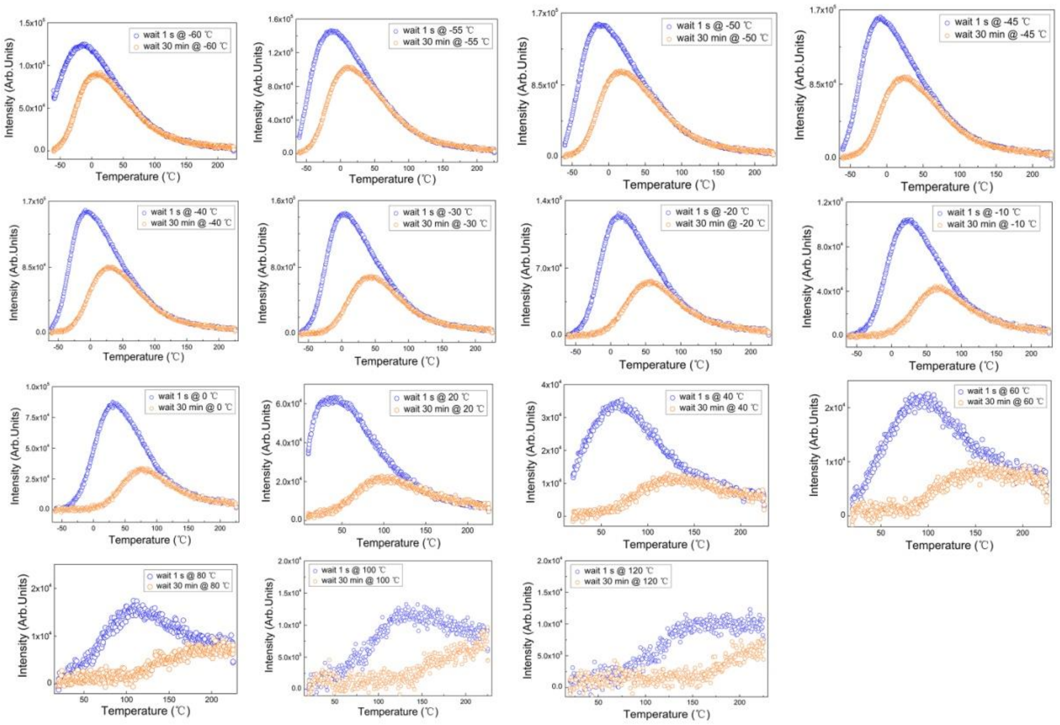

Figure S5. **TL glow curves of CaS:Eu^{2+}, Dy^{3+} (CaS) at various charging temperatures for different fading times (after irradiation for 10 min at charging temperature from -60 °C to 120 °C, fading time was chosen as 1 s or 30 min, and TL curves were collected with a constant heating rate of 30 °C min^{-1}).**

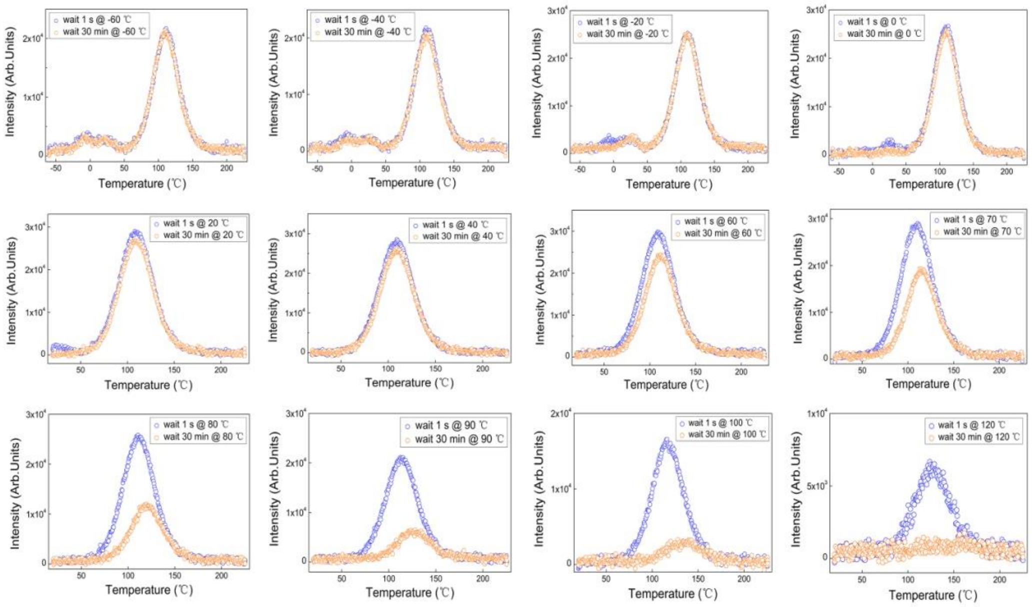

Figure S6. **TL glow curves of $LaAlO_3$:Mn^{4+}, Na^{+} (LAO) at various charging temperatures for different fading times (after irradiation for 10 min at charging**

temperature from -60 °C to 120 °C, fading time was chosen as 1 s or 30 min, and TL curves were collected with a constant heating rate of 30 °C min^{-1}).

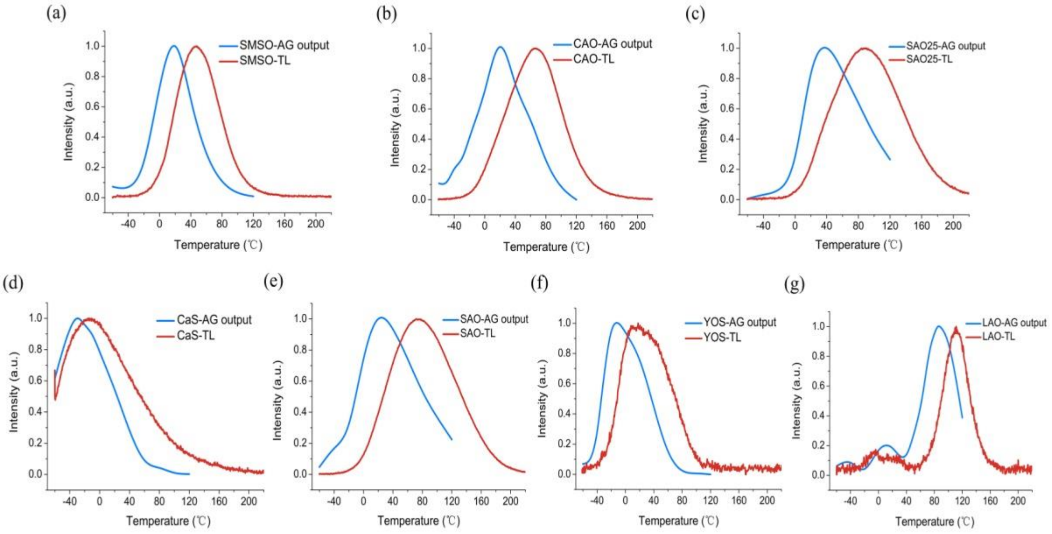

Figure S7: Comparison of the afterglow output and its TL glow curve of each LPP in (a) SMSO; (b) CAO; (c) SAO25; (d) CaS; (e) SAO; (f) YOS; (g) LAO.

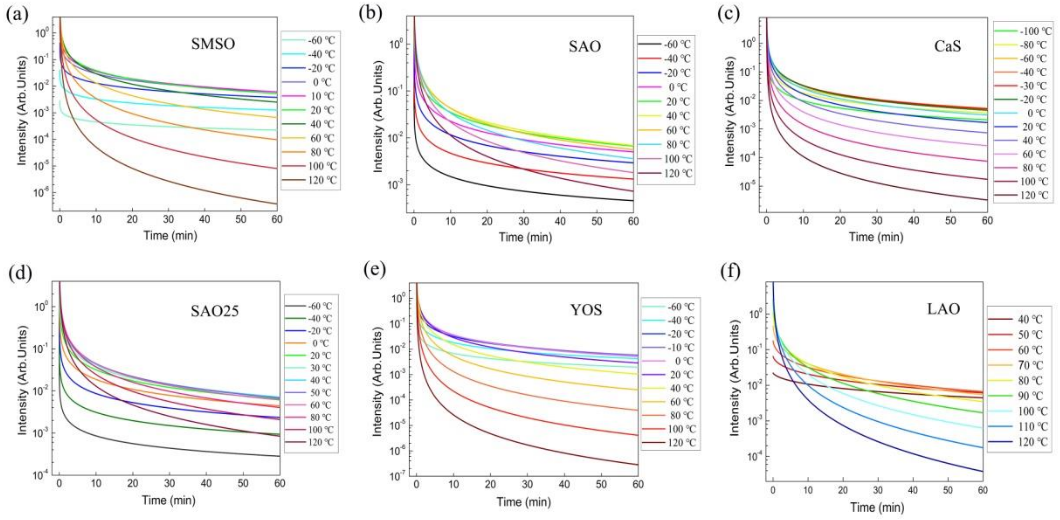

Figure S8. Prediction of afterglow profiles of the persistent phosphors (a) SMSO (b) SAO (c) CaS (d) SAO25 (e) YOS (f) LAO at different temperatures.
